# The Biological and Clinical Aspects of a Latent Tuberculosis Infection

**DOI:** 10.3390/tropicalmed7030048

**Published:** 2022-03-08

**Authors:** Nelli F. Khabibullina, Daria M. Kutuzova, Irina A. Burmistrova, Irina V. Lyadova

**Affiliations:** National Medical Research Center of Phthisiopulmonology and Infectious Diseases under the Ministry of Health of the Russian Federation, 127994 Moscow, Russia; kutuzovadm@nmrc.ru (D.M.K.); burmistrovaia@nmrc.ru (I.A.B.); lyadova@idbras.ru (I.V.L.)

**Keywords:** latent TB infection, persistence, phenotypic insusceptibility, dormant *Mtb*, anti-TB drugs, chemoprophylaxis, immunodiagnosis, microRNA, gene expression

## Abstract

Tuberculosis (TB), caused by bacilli from the *Mycobacterium tuberculosis* complex, remains a serious global public health problem, representing one of the main causes of death from infectious diseases. About one quarter of the world’s population is infected with *Mtb* and has a latent TB infection (LTBI). According to the World Health Organization (WHO), an LTBI is characterized by a lasting immune response to *Mtb* antigens without any TB symptoms. Current LTBI diagnoses and treatments are based on this simplified definition, although an LTBI involves a broad range of conditions, including when *Mtb* remains in the body in a persistent form and the immune response cannot be detected. The study of LTBIs has progressed in recent years; however, many biological and medical aspects of an LTBI are still under discussion. This review focuses on an LTBI as a broad spectrum of states, both of the human body, and of *Mtb* cells. The problems of phenotypic insusceptibility, diagnoses, chemoprophylaxis, and the necessity of treatment are discussed. We emphasize the complexity of an LTBI diagnosis and its treatment due to its ambiguous nature. We consider alternative ways of differentiating an LTBI from active TB, as well as predicting TB reactivation based on using mycobacterial “latency antigens” for interferon gamma release assay (IGRA) tests and the transcriptomic analysis of human blood cells.

## 1. Introduction

The term “latency” has two meanings: in biology, it means a dormant state of an organism when environmental conditions are not suitable for growth and proliferation, while in medicine, it is a stage of a disease when the symptoms are not yet clinically manifested [1]. An LTBI is characterized by a permanent immune response to *Mtb* antigens in the absence of any clinical manifestation of the disease [2]. During an LTBI, *Mtb* remains in an inactive state for a long time, being phenotypically insusceptible to anti-tuberculous drugs and retaining its ability to resuscitate and proliferate [3,4,5]. To achieve the global WHO goal of limiting the spread of TB by 2035, it is necessary to understand the molecular mechanisms of *Mtb* persistence and an LTBI, as well as to develop and improve methods for the LTBI diagnosis and treatment [2,6,7].

During its coevolution and long-term adaptation to humans, *Mtb* acquired the ability to remain asymptomatic (i.e., persist) in the body, even after treatment with high doses of anti-TB drugs targeting active and dividing cells [6,8,9,10]. The concept of persister existence was first introduced in 1944 to describe Staphylococcus species which survived treatment with lethal concentrations of penicillin. These cells became phenotypically insusceptible to it [10]. The phenotypic insusceptibility of persisters is a major problem in the treatment of infectious diseases [11,12]. In contrast to genetic resistance, strictly determined by the presence of genetic mutations and polymorphisms [13], phenotypic insusceptibility is caused by changes in bacterial cell physiology as adaptive reactions to stress.

## 2. Persisters and Acquired Phenotypic Insusceptibility

The nature of *Mtb* persister cells and the reason for their drug insusceptibility are not well understood so far. Usually, these cells are formed during an exponential or stationary phase, or else as a result of hypoxia, deoxyribonucleic acid (DNA) damage, or starvation. Unlike genetically resistant cells, whose resistance is coded in DNA, *Mtb* persister cells can simultaneously develop unspecific insusceptibility to many antibiotics but cannot pass this feature on to their daughter cells [14,15,16]. The persisters themselves and their non-inherited insusceptibility may arise from outer changes, such as a thickening of the cell wall [17], or due to inner cellular processes, such as pauses in DNA replication and repair by the SOS system (“Save Our Souls”, which, in the case of any bacteria, means the activation of SOS genes in response to damaged DNA) [16,18,19,20]. Persisters may also arise due to the toxin–antitoxin (TA) inhibition of translation machinery and growth arrest [21], or they may even pre-exist in the culture due to the asymmetrical ways in which *Mycobacteria* divide [22,23]. Sometimes, their colonies may have different features due to epigenetic control, as in the cases of large and small colony variants (LCV and SCV, respectively), regulated by the HupB protein [24]. However, additional reasons for both the origin and insusceptibility of persisters have been proposed. A possible mistranslation using mutated transfer RNA (tRNA) anticodons, when glutamic acid residue was replaced by glutamine and aspartic acid residue was replaced by asparagine, led to rifampicin insusceptibility, although no mutations in the β-subunit of the RNA polymerase gene were identified [25]. This could probably be explained by the formation of a protein pool that is useful for survival under stress conditions [26,27,28].

## 3. Forms of Inactive *Mtb*

After the initial contact between *Mtb* and the human body, the infection can either be completely eliminated or can progress to primary TB [29]. In people with an LTBI, the dynamic equilibrium between the host and *Mtb* is maintained by the regulation of the access to nutrients, as well as the innate and acquired immune control [30,31]. At the same time, *Mtb* bacilli that have infected the body and have not been eliminated by immune cells or anti-TB drugs persist in the body, either in granulomas, or in niches outside of them [32]. Using the Cornell mouse model as an example, it has been shown that the number of *Mtb* persisters in the mouse’s body is small (below 1000 cells per 1 mL of tissue), and, in the case of patients’ infected materials, it is almost impossible to isolate and cultivate these cells in vitro. Nevertheless, even a small number of persisters is sufficient to reactivate the infection in the body [4]. Persistent *Mtb* cells are able to survive for a long time in unfavorable conditions inside the body, maintaining their metabolism to ensure it is sufficient for a delayed revival and proliferation burst [5,33].

It has been shown that two forms of *Mtb* persisters could be differentiated. One of them is *Mtb* L-forms. They were found experimentally both in vitro and in vivo in an infected organism [33,34,35,36,37,38]. L-forms do not have a cell wall [34] and are not acid-fast. They quickly restore their metabolic activity, grow and proliferate much faster than normal *Mtb* cells, represent a variety of morphological forms [39,40] and they are insusceptible to ethambutol and streptomycin [41,42].

The second form of *Mtb* persister cells are called dormant cells [43]. Dormant cells (“viable non-cultivating cells” [5,44,45]) persist in caseous granulomas with a low vascularization and oxygen pressure, where their growth and proliferation are limited [40]. These cells can be obtained in vitro as a result of prolonged incubation at a reduced oxygen level and a low pH. They are characterized by a thickened cell wall, a decreased size, and an “ovoid” shape. Prolonged incubation (from 18 to 22 months) leads to the appearance of the thermolabile spore-like cells that are incapable of resuscitating when inoculated into a fresh medium, but can cause TB in mice when injected into the abdominal cavity [43]. Dormant *Mtb* cells are phenotypically insusceptible to isoniazid [44,45,46].

During an LTBI, the physiological and metabolic conditions of *Mtb* cells change, leading to an equilibrium between two states (active and dormant) under immune pressure [44]. In this equilibrium, the ratio of either active or dormant *Mtb* cells depends on the duration of the LTBI. The analysis of the accumulation rate of single-nucleotide polymorphisms (SNPs) in the DNA of *Mtb* isolates from cynomolgus macaques, or people, after a TB reactivation showed that a rapid accumulation of SNPs (as in active TB) occurs during the first two years of the infection (“early phase of an LTBI”), and in subsequent years (“late phase of an LTBI”) it decreases. It means that *Mtb* cells significantly reduce proliferation and become predominantly dormant eventually [47,48,49].

## 4. Regulation of *Mtb* Dormancy

Granulomas play an important role in arresting the growth and proliferation of *Mtb* cells. Inside granulomas, they are faced with hypoxia, a lack of nutrients, and high concentrations of nitric oxide [50]. However, they can survive in these conditions due to their transition to the dormant state and persisting even during the anti-TB chemotherapy [3,50,51,52]. Stress conditions in granulomas lead to the metabolic heterogeneity of *Mtb* [44,53]. Under hypoxic conditions [53,54,55]. and the pressure of immune cells (either in vitro in macrophage culture [56] or in vivo in mice [57], or guinea pigs [58], the expression of genes of the dormancy survival regulator (*DosR*) regulon is induced [41,59,60]. Depending on the virulence of the *Mtb* strain (H37Rv (“virulent”), H37Ra (“avirulent”), W-Beijing), the induction of the *DosR* regulon leads to different gene expression profiles [59,60].

The transition to dormancy is not a random process for *Mtb*. Early, middle, and late stages are distinguished in this process [61]. The dormant *Mtb* transcriptome is enriched in regulatory non-coding RNAs (ncRNAs): *Mycobacterium tuberculosis* small RNA (MTS0997 (MTS0997), MTS1338, and MTS2823. The level of MTS0997 remains constant in the early phase, but it is significantly reduced in the middle and late phases. MTS2823 accumulates in early phase cells, while the level of MTS1338 remains constant [62]. The level of MTS0997 depends on the conditions of *Mtb* growth: it increases during fasting and decreases in an acidic environment [63]. MTS0997 is functionally linked to the *Rv1264* and *Rv1265* genes. A product of the *Rv1265* gene, an adenosine triphosphate (ATP)-binding transcription factor, grows during infection and in response to an increase in cAMP concentration [64]. This factor increases the expression of Mcr11, a sRNA associated with slow *Mtb* growth and chronic infection in mice [65] in the stationary growth phase. The putative targets of MTS0997 are products of the *Rv3282*, *fadA3* (beta-ketoacyl CoA thiolase), and *lipB* (encoding for octanoyl-[acyl carrier protein]-protein acyltransferase) genes involved in lipid metabolism. The deletion of MTS0997 leads to a slower growth of *Mtb* and *M. bovis* in a lipid deficient medium. The overexpression of MTS0997 and MTS1338 leads to a slowdown in cell growth in the culture and is significantly higher in dormant “nonculturable” *Mtb* cells [62]. To summarize, the regulatory functions of ncRNA in dormant *Mtb* are as follows: mcr7 regulates virulent properties, 6C inhibits growth, MTS0997 regulates lipid metabolism, and MTS1338 controls the expression of genes in the *DosR* regulon. MTS0997 and MTS1338 are present only in the *M. tuberculosis* complex; they regulate the stress response during the phagocytosis of the pathogen in the host [63].

## 5. Latent TB Infection

The binary model of TB infection with a latent infection and an active disease has been criticized [49]. Instead, it is proposed to distinguish five discrete stages in the pathophysiological spectrum of TB infection [66]: eliminated—observed after a complete elimination of *Mtb* cells either by immunity (both innate and acquired) or after anti-TB chemotherapy, where viable *Mtb* cells do not present, although immunological signs of infection may be observed; LTBI—caused by viable *Mtb* cells, which have immunological signs of TB infection with no clinical, radiological, or microbiological evidence of TB, and will not progress to TB in the case of strong immunity; incipient—likely to progress to TB in the absence of control, but is not yet manifested either clinically, radiologically, or microbiologically; subclinical form—there are no have clinical symptoms of TB, but it already causes radiological or microbiological changes; and active TB—which causes clinical symptoms with radiological changes/microbiological signs compatible with TB. These stages can interchange with different dynamics. The increase in the disease severity usually correlates with an increase in symptoms, a low likelihood of spontaneous self-healing, a weakened or poor immune response, and an increased bacterial excretion. However, spontaneous recovery is possible at any of the stages described above [66].

Neither authors of previously published papers in the LTBI area, nor WHO recommendations, differentiate between the *Mtb* infection (as the presence of persistent *Mtb* cells in the body) and an LTBI. The definition of an LTBI, as a diagnosed immune response to *Mtb* antigens in the absence of any TB signs, is generally accepted [67,68,69]. At the same time, there is a proposal to consider an LTBI as a spectrum of various states, such as immunological reactivity, past or cured infection, lasting inactive infection, and early stage of TB. This makes it possible to not only describe the multiple stages of an LTBI, but also to personalize the LTBI treatment [49,70]. The treatment of an LTBI is necessary for people with a subclinical infection, but is not necessary when the infection has been eliminated or in case of calcifications. For example, clinical observations of 35 patients with a human immunodeficiency virus (HIV) infection, who did not receive antiretroviral therapy (ART), showed that in 10 of them (group 1) with the subclinical disease and changes in the lungs, including active nodules, infiltrates, or fibrous scars, a risk of TB development was significantly higher than in the remaining 25 (group 2) without subclinical pathology. Although, among them, 10 had normal lung parenchyma and 15 had individual nodules. Moreover, in group 1, including patients with the subclinical disease, six people did not develop TB during the entire period of the study. Thus, even with immunosuppression, the LTBI progression to TB may not occur [71,72].

## 6. Risk Factors for LTBI

According to the generalized data cited by M. Serra-Vidal et al. [73], individuals are highly likely to develop an LTBI in each of the following scenarios:

(1) If they have both a positive (IGRA) test and a positive tuberculin skin test (TST) and are in contact with TB patients;
(2) If they have a negative IGRA, but their TST size is larger than 5 mm, and they are in close contact with TB patients (when the source of infection excretes *Mtb,* and a contact was vaccinated with bacille Calmette-Guerin (*BCG)*) or when the TST size is larger than 15 mm (when the source of infection does not excrete *Mtb,* and a contact was vaccinated with *BCG*);
(3) If they have a negative IGRA and their TST size is smaller than 10 mm, but their TST size increases and becomes larger than 10 mm (the difference is not less than 6 mm);
(4) If they have a negative IGRA and positive TST (when the TST size is greater than or equal to 10 mm without *BCG* vaccination, or TST is greater than or equal to 15 mm in case of *BCG* vaccination).

People with a diagnosed LTBI have a high incidence of a TB clinical manifestation. The chance of further manifestation is higher in case of comorbidities, continuous contact with the source of infection, and a compromised immune system. However, the data on this topic are contradictory due to large-scale *BCG* vaccinations, making the interpretation of the results and prognosis complicated [74,75,76,77].

Most studies show that some high-risk factors (HIV, organ transplantation, silicosis, treatment with tumor necrosis factor-α (TNF-α) blockers, hemodialysis, and close contact with *Mtb* excreting patients) accelerate the reactivation of TB infection significantly [78,79,80]. Therefore, such patients should be regularly tested for an LTBI [78,81].

## 7. LTBI Diagnosis

Immunological methods occupy a special place in the diagnosis of an LTBI [82]. Previously used diagnostic tools were based on the results of the TST [74]. It does not allow the differentiation between an LTBI or TB [83], but it is currently widely used in some countries due to its low cost. Vaccinations with *BCG,* as well as non-tuberculous mycobacterium (NTM) infections in patients with HIV, lead to false positive results [84,85]. False negative results can be observed in immunocompromised patients receiving immunosuppressants [86,87].

Modern tests are based on the evaluation of the levels of IFN-γ production after stimulation with the recombinant ESAT-6/CFP-10 fusion protein, with the early secreted virulence factors encoded in the RDI (region of difference) of *Mtb* [7]. These tests include in vitro IGRA tests (QuantiFERON (QFT)-TB, T-SPOT.TB test, IFN-γ-inducible protein 10 (IP-10)) and in vivo TST tests (with tuberculin or a recombinant TB allergen). IGRA tests are more specific than TST: they estimate the level of IFN-γ released by T-cells after stimulation with ESAT-6/CFP-10, which are absent in *BCG* and NTM [84]. The specificity, sensitivity, and results of IGRA tests can differ from test to test for various reasons, including the patient’s age, country of origin, and gender [88,89]. Moreover, the presence of comorbidity should be considered. For example, severe diabetes mellitus in a patient led to a negative result on all tests when comparing QFT-Plus, QFT-GIT, and T-SPOT [90]. Reactions of immune memory can also cause difficulties in diagnoses, leading to false positive results [76,91]. It has also been shown that ESAT-6, which is present in the vast majority of in vitro tests in high (microgram) amounts, is, itself, capable of enhancing inflammatory responses [7], leading to ambiguous results. Thus, a combined approach is required to diagnose an LTBI, but such an approach does not yet exist [74,92].

The identification of several groups of genes responsible for different processes in the *Mtb* metabolism and life cycle, either in vitro, in animal models, or in humans, made it possible to find *Mtb* antigens that could be efficient in the discrimination of an LTBI (Table 1).

These antigens were identified as potent latency-related biomarkers for an LTBI diagnosis. Some of them were identified after modeling in vitro hypoxia [73], nutritional deprivation [93], and NO introduction [94], and, afterwards, were produced and purified as recombinant proteins in *Escherichia coli* cells. The others were found directly in *Mtb* cells, extracted from the lungs of infected mice [95]. The *Mtb* antigens were tested for their potency in the induction of interleukin production and were screened using both mice and human T-cells. Based on these, several antigen panels were developed. They included the antigens whose expressions were induced under a range of conditions: nutrient starvation (Rv0287/88, Rv0470c, Rv0640, Rv0645, Rv1221, Rv1284, Rv1980, Rv2873, Rv3614/15, Rv3616, and Rv3865), hypoxia (Rv0826, Rv0991c, Rv1221, Rv1284, Rv2007, and Rv2626c), vitamin C exposure (Rv2626c, Rv0467, Rv1221, Rv0991, Rv3615c, and Rv3616c), and intra-phagosomal infection in naive and activated macrophages (Rv0467, Rv0642, Rv0826, Rv1121, Rv1980, Rv2007, Rv2626, and Rv2873) [95].

Moreover, it was shown that *Mtb* Rpf antigens may be suitable for the screening of T-cell responses to TST-positive people. In this case, IFN-γ production was observed in response to Rv1009 (rpfB), Rv1884c (rpfC), Rv2450c (rpfE), and, to a smaller degree, Rv0867c (rpfA) [96]. Among them, Rv0867c and Rv2389c (rpfD) were predicted to be secreted proteins, thus allowing immune cells to recognize them efficiently. A further investigation of these two antigens showed that Rpf-specific memory CD4 and especially CD8 T-cells can provide long-term protection for people with an LTBI that does not progress to TB [97]. In another study, Rpf antigens (Rv0867c, Rv2389c, Rv2450c, Rv1009, and Rv1884c) showed their effectiveness for the identification of active TB. A combination of *Mtb*-specific ESAT-6/CFP-10, Rv0867c, and Rv2624c had accurately identified 73% of TB patients and 80% of the non-TB cases after cross-validation [98].

Rv1733c, a putative membrane protein, was shown to be preferentially recognized by T-cells from people with an LTBI, compared to TB patients [99]. Antigens Rv3873, Rv3878, and Rv3879c showed their prognostic efficiency in a study of 846 children exposed to TB. The dynamics of the TST conversion over six months, as well as their clinical outcomes two years after the study, correlated with the results when those antigens were used [100].

The *DosR*-controlled antigen—alpha-crystallin (Rv2031)—was shown to enhance the efficiency of an LTBI diagnosis in a TB endemic setting via a strong increase in IFN-γ, TNF-α, and interleukin-10 levels. The increased levels of these cytokines were suggested as biomarkers of an LTBI [101]. The antigens Rv3133c, Rv2031c, Rv1733c, Rv2029c, Rv2626, R2628, Rv0475, Rv0867c, Rv1009, Rv1884c, Rv2389c, and Rv2450c, produced under the *DosR* regulon, were purified as recombinant proteins and were tested for differentiating people with an LTBI from people with TB in Europe, Africa, South America, and India. In all cohorts, these antigens mostly stimulated a response in people with an LTBI, compared to patients with TB (either active or treated) [102]. The Rv2626c, Rv2627c, Rv2628, Rv2031c, and Rv2032 antigens were used for the evaluation of IFN-γ levels in day 1 (short-term response) and day 7 (long-term response) assays during the different stages of TB. QFT-GIT-positive people with a remote infection had a fivefold higher response to Rv2628, which could be explained by the immune-mediated protection against TB, and, thus, can be used for distinguishing between an LTBI and a recently acquired infection [103]. A two-hit assay, combining the use of ESAT-6/CFP-10 with additional antigens (Rv2628, Rv1733, Rv2031, and Rv3407), allowed for the identification of the LTBI possessors who were not responsive in the QFT assay [104].

Rv0847, produced under the control of the copper inducible five-locus regulon, is specific to virulent mycobacterial species. Its role was shown to be the ability to sense the host macrophage environment, and it might be crucial to *Mtb* survival under hypoxia and nonreplicating conditions [105]. The antigen had shown a substantial induction of IFN-γ levels in the LTBI patients after a long-term stimulation (7 days) and a certain IFN-γ level in infected people after a short-term stimulation (overnight) [73].

Using Rv2659 alone did not detect any difference between TST-negative people and TST-positive healthy people. However, a significant difference was shown between TST-positive people and TB patients. Rv2660 did not reveal a statistical difference between TST-negative controls and TST-positive healthy people, whereas the difference between the TST-negative controls and TB patients was significant [106]. Rv1985c was specifically recognized at the levels of cellular and humoral responses from both the TB and LTBI groups, compared with healthy controls. The addition of Rv1985c increased the sensitivities of ESAT-6, CFP-10, and an ESAT-6/CFP-10 combination in detecting TB from 82.1% to 89.2% (*p* = 0.125), 67.9% to 87.5% (*p* < 0.001), and 85.7% to 92.9% (*p* = 0.125), respectively [107].

The so-called “latency antigens” of *Mtb* showed their potent efficiency in identifying people with an LTBI and distinguishing them from healthy controls or active TB patients based on the IFN-γ production levels. Moreover, in some cases, they were efficient even in differentiating between an LTBI and a recently acquired *Mtb* infection and, thus, could be considered as novel candidates to expand the IGRA test antigen panel.

## 8. Chemoprophylaxis

The TB process is a dynamic relationship between *Mtb* cells (actively dividing and dormant) and the immune cells [44]. In an LTBI, the number of actively dividing cells is small, but it is sufficient to reactivate the infection. Chemoprophylaxis (CP) is active against actively dividing *Mtb*, preventing the reactivation of TB [44,108]. Currently, various approaches are used for chemoprophylaxis around the world. The use of first-line anti-TB drugs, rifamycin/rifapentine or isoniazid, is widespread [50,109]. This approach to TB chemoprophylaxis appeared in the 1950s and was based on the observation that the treatment of children with isoniazid prevents the development of TB symptoms. The effectiveness of this approach was shown in a controlled clinical study that involved 2750 children with asymptomatic primary TB or recent TST conversions: the development of TB decreased by 94% during the year of the LTBI treatment and by 70% in the next 9 years [92,110]. If the source of the infection has drug resistant TB, chemoprophylaxis should include the anti-TB drugs to which the isolate in question is sensitive. In a prospective cohort study of South African children after household contacts with patients with multidrug-resistant TB (MDR-TB), sensitive to fluoroquinolones, the prophylactic effectiveness of the use of fluoroquinolones alone, or in combination with ethambutol, ethionamide, cycloserine, or para-aminosalicylic acid, was shown [110]. The effective duration of the LTBI treatment for MDR-TB contacts is unknown, but a regimen of 6 to 12 months at standard dosages is used widely [92,110].

Chemoprophylaxis in adults should focus on high-risk groups (HIV patients, organ transplant recipients, silicosis patients, and contacts). The effectiveness of isoniazid monotherapy has been shown for the first two groups [73]. At the same time, for patients with an HIV infection and an LTBI, the combination of ART with isoniazid prevented the progression of TB [80]. Despite the effectiveness of isoniazid, daily, for 6–12 months, in preventing 60–90% of TB cases, this regimen is limited by poor tolerance, a long duration of treatment, and low patient adherence [111]. It has been shown that, on average, with a 9-month regimen of isoniazid taken daily, patients adhered to the prescribed therapy for only 30 days, and then quitted it voluntarily [112].

To increase the effectiveness and reduce the duration of chemoprophylaxis, various schemes are used in world practices. The effectiveness of therapy with isoniazid and rifapentine once a week for 12 weeks has been shown for homeless people [111]. A study conducted in Taiwan from 2014 to 2016 showed that 12 weeks of treatment with isoniazid + rifapentine (once a week) is more effective than 9 months of treatment with isoniazid daily [113]. The PREVENT TB trials compared the same regimens for the same duration, but with a direct observation of the drug intake, and they received similar results [111]. An open randomized multicenter controlled trial involving nine countries compared two regimens for chemoprophylaxis in adults: 4 months of rifampicin daily, versus 9 months of isoniazid daily. A shorter rifampicin regimen has been confirmed to be associated with improved adherence to treatment [114]. In an open-label, randomized, multicenter pilot study in London from 2015 to 2017, two regimens were shown to be equally effective: self-administered rifapentine/isoniazid weekly, or rifampicin/isoniazid daily for 12 weeks. This suggests the possibility of reducing chemoprophylaxis side effects and increasing patient adherence to anti-TB drugs [115]. Table 2 shows a comparison of prophylactic regimens.

An equally important criterion in the LTBI treatment is the total cost of chemotherapy. One study in Australia analyzed the cost of two LTBI treatment regimens: 9 months of isoniazid monotherapy and 12 weeks of isoniazid/rifapentine regimens. The cost of one completed course of treatment was AUD 601 for the first regimen– and AUD 511 for the second [121]. Thus, from an economic point of view alone, the isoniazid/rifapentine weekly regimen may be beneficial to the LTBI therapy. This provides financial encouragement for the population in order to improve treatment coverage [110]. The current LTBI treatment includes mostly anti-TB drugs that target metabolically active and dividing bacilli. The only exclusion is pyrazinamide, which is active against nongrowing *Mtb* persisters, and helps to shorten the anti-TB therapy duration significantly [122]. The combination of rifampicin + pyrazinamide, although shown to be very effective against an LTBI, led to severe side effects and was not recommended by the WHO for the LTBI treatment [118,123,124,125,126,127].

However, new anti-TB compounds are under exploration. They are targeted at blocking the enzymes that remain active during the dormant state of *Mtb* cells: alanine dehydrogenase, isocitrate lyase, cysteine synthase CysM, adenosine-5′-phosphosulfate reductase, DevS and DosT oxygen sensors involved in dormancy response, lysine ε-aminotransferase, enoyl-acyl carrier protein reductase (InhA), decaprenyl-phosphoryl-ribose 2′-epimerase (DprE1), mycocyclosin synthase (Cyp121), and extracellular zinc metalloprotease 1 (Zmp1). Details of the above-mentioned compounds targeting nonreplicating *Mtb* cells, a description of their chemical structures, and the possible modes of action are provided in the review by Campaniço et al. “Addressing latent TB: new advances in mimicking the disease, discovering key targets, and designing hit compounds” [50].

Another possible direction of the anti-TB drug design is preventing TB reactivation via inhibiting resuscitation promoting factors (Rpfs), which are hydrolases with muralytic activity that are able to cleave the cell wall of dormant *Mtb* cells, thus promoting their resuscitation. The *Mtb* genome contains five genes encoding sequences for the Rpf A-E proteins [128]. Since these proteins promote *Mtb* resuscitation and TB reactivation, they are of great interest as potential targets for the development of anti-TB drugs aimed at treating LTBIs and preventing TB reactivation [129]. These potential anti-TB drugs are nitrophenylthiocyanates (NPTs), capable of inhibiting the biological and enzymatic activities of Rpfs. Among the NPTs, two components—3-benzoylphenyl thiocyanate and 4-benzoylphenyl thiocyanate—showed the maximum activity in inhibiting the Rpf-mediated peptidoglycan hydrolysis and in the resuscitation assay of the dormant *Mtb* bacilli [130].

## 9. The Controversy of LTBI Diagnosis, Adaptive Immunity, and Chemoprophylaxis Prescription

An essential component of the WHO End TB Strategy is TB prevention by identifying and treating people with an LTBI based on positive immunological tests. In countries with a high TB burden, the LTBI treatment is the most important strategy for TB control. Nevertheless, a point of view, that the attention to LTBIs is exaggerated, also exists [1]. About 10 million new TB cases are diagnosed each year, but there are no published data on differentiated primary infections. Numerous longitudinal studies show that TB often manifests in the first two years after infection [131]. Therefore, in order to decrease the TB reservoir, attention should be paid not to chemoprophylaxis, but to the treatment of TB patients and their contacts.

Although immunological tests are commonly used to identify individuals with an LTBI, they only reflect the presence of immune responses to *Mtb* antigens. However, the immune response to *Mtb* antigens will take place even after all live *Mtb* bacilli have been eliminated either by immunity or due to their treatment with anti-TB drugs [76]. If the immunoreactivity to TB is a marker of an LTBI, then immunological reactions should become negative after treatment. However, in practice, this is not always the case. A large-scale study in the United States showed that although the intake of isoniazid in people immunoreactive to *Mtb* for one year reduced the incidence of TB by 60–70% over the next 9 years, the TST of these people remained positive [1]. Another study showed that if people tested positive a year before treatment, they would remain so after treatment. At the same time, 50% of people who were immunoreactive for less than a year, either went on to test negative, or the size of their TST levels decreased [132]. Similar results were obtained in another study: those who were immunoreactive for a longer period of time before their treatment with isoniazid were more likely to remain immunoreactive after treatment [133].

Moreover, the use of IGRA tests to diagnose the effectiveness of treatments does not shed light on the cause of the immune response. It remains uncertain as to whether it is caused by the persistence of *Mtb* cells in the body, or by its stimulation with mycobacterial antigens, while viable bacilli have long been eliminated. The results of testing with QFT-TB, before starting chemoprophylaxis in people with a suspected LTBI in a country with low TB endemicity, was positive in 30.8% (148/481) of the total number of subjects, in 66.9% (111/166) of people who moved from TB-endemic countries, in 71.4% (20/28) of those who were previously treated for TB, and in 100% (15/15) of those diagnosed with active TB. These people were given chemoprophylaxis, and then the QFT-TB test was repeated. In 35/40 (87.5%) and 22/26 (84.6%) of people, the QFT-TB test was positive after 3 and 15 months of treatment, respectively [134]. In another study, the use of QuantiFERON-TB Gold In-tube (QFT-IT) and T-SPOT.TB showed a reduction in positivity rates after 6 months of chemoprophylaxis completion. At the same time, QFT-IT was less likely to show positive results, compared to T-SPOT.TB: 46% and 79% of people tested positive after completing a course of chemoprophylaxis, respectively [135]. In one more trial, QFT-GIT was used after the completion of 4 months of chemoprophylaxis in people with previously positive results for both TST and QFT-GIT. The TST was positive in 67.7% of people, while the QFT-GIT was positive in 56.7% out of 214 people. The QFT-GIT was positive in 77% (97/126) of people with a positive TST. Chemoprophylaxis was completed by 81 people. Among 74 of them, IFN-γ levels decreased in 97.3% of people (72/74), and a positive-to-negative reversion was shown in 31 people (41.9%) [136].

These results are consistent with the development of a strong immunological memory, which is enhanced by prolonged exposure to the antigen [91], but it is still not clear if the chemoprophylaxis, with its possible severe side effects, is indeed necessary for all LTBI-suspected people, as well as at what time after TB exposure it should be started, and whether it can really protect people from TB reactivation during their lifespan.

However, despite the facts above, computer modelling has shown that if at least 8% of people with an LTBI received treatment annually, the total global incidence of TB would be fourteen times lower by 2050 compared to 2013, even in the absence of additional measures [110].

## 10. Transcriptomic Analyses as a Promising Tool for LTBI and TB Reactivation Diagnoses

Transcriptomic analyses make it possible to determine the individual gene expression signatures of the host in response to a pathogen. It has already been shown that the levels of gene expressions change in diseases of various etiologies, including psoriasis [137], parasitic diseases [138], and Parkinson’s disease [139]. This analysis can differentiate between active TB and pneumonia or lung cancer, but still there are no clear criteria for distinguishing between active TB and sarcoidosis [140].

This method has already shown to be both high in specificity and sensitivity for differentiating between active TB and LTBIs in adults. It was observed that changes in the expression levels of the *CXCL10*, *ATP10A*, and *TLR6*, as well as the *DOCK9*, *EPHA4*, and *NPC2* genes from peripheral blood samples, incubated with PPD, significantly differed in patients with active TB, people with an LTBI, and healthy individuals from England, Africa, and Brazil [141,142]. Moreover, it has been shown that the elevated expression level of *NPC2* in one of the patients who did not receive chemoprophylaxis subsequently led to the development of active TB, but after treatment, it decreased significantly [111]. In 2018, a meta-analysis provided a panel of genes: *CXCL10*, *DUSP3*, *FCGR1A*, *GBP5*, *SEPT4*, *ANKRD22*, *BATF*, *FCGR1B*, *FCGR1C*, *GAS6*, *GBP1*, *GBP6*, *LHFPL2*, *S100A8*, *SCARF1*, and *SERPING1*, which were the most strongly associated with TB [143]. Then, in 2020, the “RISK6”, which is a six-gene transcriptomic signature of TB reactivation, was introduced [144]. It contained a panel of genes from various cohorts of patients from South Africa, Peru, and Brazil. This panel included six genes (*SERPING1*, *TRMT2A*, *GBP2*, *SDR39U1*, *FCGR1B*, and *TUBGCP6*) whose expression levels demonstrated the highest predictive power for the risk of TB reactivation.

The products of the *CXCL10*, *DUSP3*, *FCGR1A*, *GBP5*, *SEPT4*, *ANKRD22*, *BATF*, *FCGR1B*, *FCGR1C*, *GAS6*, *GBP1*, *GBP6*, *LHFPL2*, *S100A8*, *SCARF1*, and *SERPING1* genes were most frequently involved in immune responses against pathogens:

*CXCL10* encodes a proinflammatory chemokine of the Cysteine X Cysteine (CXC) family, which is involved in a wide range of processes, including leukocyte chemotaxis, the differentiation and activation of peripheral immune cells, and the regulation of cell growth, apoptosis, and the modulation of angiostatic effects;

*DUSP3* encodes a dual specificity phosphatase 3 (which dephosphorylates phosphoserine and phosphotyrosine residues), which negatively regulates mitogen-activated protein kinases;

*FCGR1A* (or *CD64*), *FCGR1B*, and *FCGR1C* (pseudogene) encode high-affinity receptors for fragments crystallizable (Fc) of immunoglobulin-γ, as well as playing a role in the activation of innate and adaptive immunity, and they are present in monocytes and macrophages;

*GBP1*, *GBP5*, and *GBP6* encode guanylate-binding proteins involved in the activation of the inflammasome and autophagolysosome assemblies in response to a bacterial infection;

*SEPT4* encodes septin, a guanosine triphosphate hydrolase (GTPase) that is a part of filaments, and plays a role in the recognition of pathogen cells;

*ANKRD22* encodes a protein with an unidentified function containing an ankyrin repeat domain;

*BATF* encodes a transcription factor of the Activator protein 1 (AP-1) family, which controls the differentiation of immune cells;

*GAS6* encodes a specific growth arresting protein, and is a ligand of Axl receptor tyrosine kinase, tyrosine-protein kinase receptor TYRO3, and tyrosine-protein kinase MER, as well as inhibiting innate cellular immunity;

*LHFPL2* encodes a protein of the tetraspan transmembrane family responsible for cell adhesion, motility, activation, and proliferation;

*S100A8* encodes a protein of the calprotectin family that regulates immune responses and inflammation, and this gene also activates the nicotinamide-adenine dinucleotide phosphate (NADP) oxidase complex, inducing its assembly;

*SCARF1* encodes a scavenger receptor family protein that mediates the binding and degradation of acetylated low density lipoproteins;

*SERPING1* encodes an inhibitor of plasma C1 proteases responsible for the inhibition of the complement system.

Some researchers believe that the correct transcriptomic analysis should include the study and comparison between the gene expression levels of the individual immune cell populations: mononuclear cells [145], neutrophils [146], and CD4 T-cells [147].

## 11. Concluding Remarks

The goals of the WHO TB strategy are to reduce TB mortality by up to 95%, and morbidity by up to 90%, by 2035. People latently infected with *Mtb* are the main reservoir of TB; therefore, their identification and treatment is an important strategy. It can effectively reduce the risk of TB and the number of potential sources of infection in the future [84,112]. Therefore, it is necessary to identify all cases of the disease and introduce chemoprophylaxis for people with an LTBI [113] more intensively. The lack of a gold standard for an LTBI diagnosis is still a problem to be resolved. Modern immunodiagnostic tests cannot fully differentiate between an LTBI, active or past TB, or the prediction of the progression of an LTBI to TB. This means that the true prevalence of an LTBI remains unknown, and estimations of the sensitivity and specificity of diagnostic tests are unreliable. However, “latency antigens” may be used for the expansion of IGRA tests, in order to differentiate between an LTBI and TB. The only registered anti-TB *BCG* vaccine does not provide the adequate prevention of pulmonary TB in adolescents and adults, allowing the infection to spread [148]. It is, therefore, crucial to gain a fundamental understanding of both *Mtb* cell and immune cell physiology, as well as to shed light on their states during an LTBI and the reactivation of the infection.

## Figures and Tables

**Table 1:** *Mtb* antigens used to assess in vitro IFN-γ production for LTBI diagnosis.

Antigens	Loci Function	In Vitro Assessment
PPD (purified tuberculin derivative)	Loci not studied; functions are diverse	IFN-γ production and other tests (especially widely used in TST)
ESAT-6, early secretory antigen/CFP-10, antigen culture filtrate	*RD1*	IFN-γ production and other tests (level of T-lymphocytes producing IFN-γ)
Rv1733	*DosR*	IFN-γ production
Rv1471, Rv2662, Rv3862	Loci (antigens) of reactivation	IFN-γ production
Rv2389	*Rpf* locus (antigens), with exit of mycobacteria from dormant state	IFN-γ production
Rv2660	Loci (antigens) metabolism in conditions of reduced nutrient intake	IFN-γ production
Rv0244, Rv1909, Rv2913	Loci (antigens) with stress induced *Mtb* functions	IFN-γ production
Rv0847, Rv0967, Rv1806, Rv2380M, Rv2435n, Rv2642	Loci (antigens) expressed by *Mtb* in vivo (IVE)	IFN-γ production
α-crystallin (16kDa-R2031c, hspX)	“Latency”	IFN-γ production
Rv3407	“Latency”	IFN-γ production
Rv2660, Rv2659	RD11, locus (antigens) of aging	IFN-γ production
PPD, CFP-10, ESAT-6, Rv3879c, Rv3878, Rv3873, α-crystallin	Various, including *DosR*	IFN-γ production

**Table 2:** Comparison of treatment regimens for the treatment of LTBI in world practice.

Anti-TB Drug	Treatment Duration	Benefits of Treatment	Disadvantages of Treatment	Adverse Events	Reference
Rifampicin	4 months, daily rifampicin intake	Greater adherence and fewer side effects	Development of side effects	Hepatotoxicity; immunoallergic reactions: minor (a cutaneous, gastrointestinal, or flu-like syndrome) or major (hemolytic anemia, shock, or acute renal failure); discoloration of body fluids	[114] [116] [117]
Isoniazid	6–12 months, daily intake	Isoniazid prophylaxis provides an additional protective effect of ART	Treatment duration, low patient adherence, development of side effects	Hepatotoxicity, estimated at 1 to 4%, occurring within the first few months after starting treatment; peripheral neuropathy, which can be prevented by the addition of vitamin B6 (pyridoxine); dermatitis and lupus syndrome	[83,88,115,117]
Rifampicin + pyrazinamide	2 months, daily intake	Greater adherence, given the duration of therapy	Development of undesirable phenomena. Not recommended by WHO	Severe hepatotoxicity; increased uric acid levels, joint pain	[78,118]
Isoniazid + rifapentine	Once a week for 12 weeks	Short duration of the regimen, low incidence of side effects	Development of side effects	Hepatotoxicity rarely occurs Rifampicin is a potent inducer of the hepatic CYP450 system in the liver and intestine. It also induces increasing hydrazine production via isoniazid hydrolase (especially in slow acetylators) Rarely: pyrexial syndrome, renal failure, precipitous thrombocytopenia, epistaxis, and bleeding of the tongue and lips	[116,117] [119] [120]
